# BacHbpred: Support Vector Machine Methods for the Prediction of Bacterial Hemoglobin-Like Proteins

**DOI:** 10.1155/2016/8150784

**Published:** 2016-02-29

**Authors:** MuthuKrishnan Selvaraj, Munish Puri, Kanak L. Dikshit, Christophe Lefevre

**Affiliations:** ^1^Institute of Microbial Technology (CSIR), Sector 39A, Chandigarh 160036, India; ^2^Fermentation and Protein Biotechnology Laboratory, Department of Biotechnology, Punjabi University, Patiala 147002, India; ^3^Centre for Chemistry and Biotechnology, Deakin University, Geelong, VIC 3217, Australia

## Abstract

The recent upsurge in microbial genome data has revealed that hemoglobin-like (HbL) proteins may be widely distributed among bacteria and that some organisms may carry more than one HbL encoding gene. However, the discovery of HbL proteins has been limited to a small number of bacteria only. This study describes the prediction of HbL proteins and their domain classification using a machine learning approach. Support vector machine (SVM) models were developed for predicting HbL proteins based upon amino acid composition (AC), dipeptide composition (DC), hybrid method (AC + DC), and position specific scoring matrix (PSSM). In addition, we introduce for the first time a new prediction method based on max to min amino acid residue (MM) profiles. The average accuracy, standard deviation (SD), false positive rate (FPR), confusion matrix, and receiver operating characteristic (ROC) were analyzed. We also compared the performance of our proposed models in homology detection databases. The performance of the different approaches was estimated using fivefold cross-validation techniques. Prediction accuracy was further investigated through confusion matrix and ROC curve analysis. All experimental results indicate that the proposed BacHbpred can be a perspective predictor for determination of HbL related proteins. BacHbpred, a web tool, has been developed for HbL prediction.

## 1. Introduction 

Hemoglobin, the oxygen carrying protein first discovered in humans, was thought to be present exclusively in eukaryotes, but this old paradigm changed when a Hb-like (HbL) protein was discovered in the bacterium* Vitreoscilla* [1]. HbL proteins have now been detected in all kingdoms of life. The recent upsurge in genome data has indicated that HbL proteins may be widely distributed among bacteria and may perform a myriad of functions apart from simple oxygen binding and storage [2]. HbL proteins found in bacteria display large variations in their amino acid sequences and structural organization. However, the basic architecture of the globin fold and amino acid residues needed for maintaining a common structural organization are conserved throughout the globin family. Three distinct structural organizations have been observed in bacterial hemoglobin: single domain HbL proteins exhibiting a classical globin-like fold, truncated HbL proteins displaying truncation in their helical structure, and chimeric HbL proteins where the globin domain is integrated with other domains having different functions [3]. Functionally, the chimeric HbL proteins (flavohemoglobin) have been further classified into three groups: (1) globin domain with only distant similarity to the FAD-domain (FAD—insignificant according to Pfam), (2) flavohemoglobin proteins containing additional cytochrome reductase domain at their C-terminus and a FAD/NAD-binding FR-type domain, and (3) globin with FAD/NAD-binding FR-type domain.

It is interesting to note that multiple HbL proteins may be present in one bacterium [4, 5]. At present, over 7 million bacterial protein sequences are available on the NCBI website, but only 1447 bacterial HbL proteins have been identified through experimental or bioinformatics analysis so far (all bacterial sequences annotated with the keyword “hemoglobin” in NCBI). Among these, only a small number of HbL proteins have been experimentally validated. It is thus likely that many HbL encoding genes have not yet been discovered. Looking into the diverse functions of HbL proteins in bacteria (e.g., oxygen metabolism, environmental stress management, virulence, signal transduction, and redox regulations) [6, 7], it is important to identify HbL proteins in bacteria in order to better understand the role and functionality of this important class of proteins. Hence, a facile online prediction system to detect the occurrence of new HbL proteins in bacteria sequence data was needed.

During the last decade, the number of known protein structures has increased enormously due to rapid advancement in structural genomics, which has inspired the development of various prediction tools for the characterization of novel protein sequences [8]. The SVM approach has been successfully applied to predict peptide features and to various types of protein classification/prediction methods including structure and function prediction. For example, the SVM approach has been used to predict antibacterial peptides and secretory proteins and it was shown that SVM performed generally better than artificial neural networks (ANN) [9, 10]. SVM based methods have been originally developed to predict the subcellular localization of human proteins [11], structural classes, and DNA-binding proteins [12, 13]. In addition, various prediction methods based on position specific scoring matrix (PSSM) have been described in the literature [14, 15].

Previously, there have been several SVM based methods proposed in the literature to deal with functional proteins, such as G-protein coupled receptors, RNA-binding proteins, and DNA-binding proteins [16–20]. To the best of our knowledge, in silico prediction methods are not yet available for HbL proteins such as bacterial HbL proteins using machine learning approaches. In the past, bioinformatics studies have classified and assigned sequences to particular oxygen-binding proteins using SVM, but this does not include bacterial HbL proteins [21]. Hence, an analysis of computer-based prediction is needed to identify new bacterial HbL proteins.

Finally, a web server for the prediction of bacterial HbL proteins has also been made freely available that implements and demonstrates various SVM models. This server allows users to submit one-letter amino acid sequence in the text area provided in the submitted form. The sequence should be in plain text without any header format. It takes a single sequence as input and predicts the corresponding HbL subclass/family protein.

## 2. Results and Discussion

Support vector machines have been used to develop prediction methods for several functional classes of proteins, such as subcellular localization, DNA-binding proteins, and RNA-binding protein recognition sites [22]. We have developed a series of SVM modules to predict HbL proteins with high accuracy. In this study, we address two types of inquiry: (1) the discrimination of potential HbL from non-HbL proteins sequences with their subclassification into their three subfamilies and (2) the assignment of domains into five distinct HbL proteins domains. SVM modules have been developed for the prediction of HbL proteins using amino acid composition (AC) and dipeptide composition (DC), PSSM, and MM profiles and hybrid approach (AC + DC). Performances were analyzed to identify methods of high prediction accuracy. The method described here will assist those who are working on bacterial HbL proteins.

### 2.1. Composition Analysis

Supplementary Figure-1a (see Supplementary Material available online at http://dx.doi.org/10.1155/2016/8150784) shows a comparison of the amino acid composition of known HbL proteins to the composition of sequences from the randomly picked set of proteins (non-HbL), as described in methods. Overall the amino acid composition distribution of HbL proteins is similar to other proteins; we have calculated the median scores between HbL and non-HbL proteins, finding that residues Ala(A), Glu(E), and His(H) are 0.5% more in HbL proteins. The residues Ser(S) and Thr(T) are present by more than 0.5% in non-HbL than the HbL proteins.

The amino acid compositions of each HbL protein subfamily are shown in Supplementary Figure-1b. It can be seen that most amino acid residues are evenly distributed in similar proportions. However, certain types of amino acid residues present variable abundance between classes. The amino acid distributions are within the HbL subfamilies, sHb class Lys(K) is most abundant amino acid present in more than 8%, and Ile(I) and Asn(N) are having more 3% than the other HbL subclasses according to their median scores. In flavoHb, Ala(A), Gly(G), Gln(Q), Ser(S), Thr(T), and Val(V) residues are having less than 2% and it is better than the other two subclasses. In trHb, residue Arg(R) is the most abundant residue of presence of above 2%; residues Asp(D), Phe(F), Pro(P), and Trp(W) are having presence of less than 2% and it shows to be better than the other HbL class according to median scores.

### 2.2. Prediction of HbL Proteins Using AC, DC, PSSM, and MM Profile

In order to discriminate HbL proteins from other protein sequences, we developed and evaluated the performance of SVM models based on amino acid composition (AC) and dipeptide composition (DC), PSSM, and MM profiles. We systematically calculated the accuracy, sensitivity, specificity, and MCC; the performance results are shown in Table 1. In this study, we have chosen the default cutoff 0.0 which shows the best MCC. The above 0.0 (negative) thresholds also predict the HbL and non-HbL correctly. Single amino acid composition (AC) models resulted in maximum accuracy of 86.14% with MCC 0.82. Similarly, SVM models developed from dipeptide composition (DC) achieved a maximum accuracy of 83.02% with MCC 0.78. The PSSM profile based prediction accuracy was 90.20% with MCC 0.89. Further, the MM residues profile achieved 86.28% accuracy with 0.83 MCC. The best overall sensitivity (SN) and specificity (SP) were achieved from all approaches (AC, DC, PSSM, and MM); the detailed results are shown in Table 1. The PSSM profile achieved the maximum accuracy (90.20%) and sensitivity (97.76%) with high confidence MCC (0.89), over all developed modules. In the fivefold cross-validation test, the average accuracy and standard deviation (SD) were calculated in each case (all approaches (AC, DC, PSSM, and MM), HbL classification, and all individual domains) shown in Supplementary Table-1 and Supplementary Table-2.

### 2.3. Classification of Bacterial HbL Proteins into Subfamilies

For each prediction method, three additional SVM modules were developed to classify HbL protein sequences in each of the three subfamilies (single domain, chimeric flavodomains, and truncated Hbs). In the classification studies, one class was used as a positive set and the remaining set was considered as negative; this has been repeated for all classes. The accuracy of the SVM prediction modules was estimated by 5-fold cross-validation and the results are listed in Table 1. In this case, single amino acid composition (AC) models resulted in accuracies of 94.96%, 96.46%, and 85.26% with MCC of 0.97, 0.95, and 0.89 for single (sHb), flavoHb, and trHbs, respectively. Dipeptide composition (DC) models achieved a maximum accuracy of 83.23%, 87.50%, and 78.17% with the MCC of 0.91, 0.80, and 0.83, respectively. With PSSM the maximum accuracies were 95.05%, 95.05%, and 87.97% with MCC of 0.97, 0.93, and 0.92, while the maximum accuracies of MM profile based predictions were 94.87%, 96.46%, and 85.07% in 0.97, 0.95, and 0.89 MCC (Table 1). In the classification PSSM modules also show the maximum accuracy, when compared to other approaches. A perfect classification method should have the sensitivity scores close to 100%. Referring to our HbL classification (Table 1), the sensitivity rate of all modules shows 100% or nearly 100%. In case of specificity, the average scores are 91.46%, 82.12%, and 78.97% for sHb, flavoHb, and trHb, respectively.

### 2.4. Classification of HbL Proteins Subfamilies into Subgroups (Domains)

To evaluate further the performance of HbL classification by SVM, modules were trained on subsets of sequences representing the different HbL protein domain subgroups. The overall detection of HbL protein with the combination of modules resulted in maximum accuracies of 91.88%, 89.65%, 83.68%, 94.96%, and 85.26% in AC, 78.09%, 87.78%, 77.05%, 83.23%, and 78.17% in DC, 84.05%, 91.04%, 82.74%, 95.05%, and 87.97% with PSSM, and 93.28%, 89.17%, 89.74%, 94.87%, and 85.07% with MM for flavoglobin (NAD-insignificant), flavoglobin-cyto-FAD/NAD, flavoglobin-FAD, single and trHb domain, respectively, as shown in Table 2. It can be observed that the SN values indicated in Table 2 are much lower than in Table 1, but the specificity value is not worse. In the average classification the SN value is 71.86% in all fHb in domain classification, the individual class shows that the average SN was 72.50%, 98.72%, and 49.23% for flavoglobin (FAD-insignificant), flavoglobin-cyto-FAD/NAD, and flavoglobin-FAD, respectively. Due to the close functional relationship of flavoglobin-FAD with the other two subclasses, the average SN rate is low.

### 2.5. Hybrid System (Combination of AC and DC Profiles)

We also tried the hybrid system, which is the combination of amino acid composition (AC) and dipeptide composition (DC) profiles. With this prediction strategy the highest accuracy was achieved: 85.21%, MCC 0.81 in HbL versus non-HbL proteins. In classification, the accuracy was 91.51%, 90.29%, and 80.03% and MCC was 0.95, 0.84, and 0.85 for sHb, flavoHb, and trHb proteins, respectively (Table 1). In the HbL proteins domain prediction, the highest accuracy was also achieved with 91.51%, 82.00%, 76.77%, 89.00%, and 80.03% and MCC was 0.95, 0.62, 0.37, 0.85, and 0.85 of sHb-globin, flavoglobin, flavoglobin-FAD-binding domain, flavoglobin-FAD/NAD-cytochrome reductase domain, and truncated hemoglobin (globin-like globin-FAM-2 domain) (Table 2).

### 2.6. Confusion Matrix and Prediction Graph Analysis

**Figure 1:**
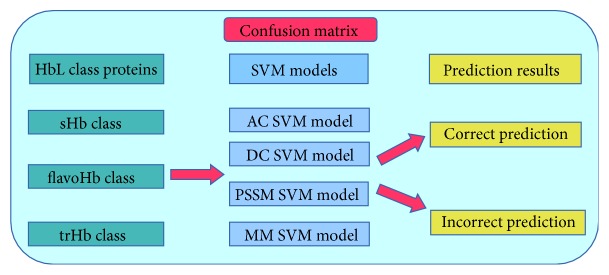
Confusion matrix system of bacterial Hbs (single domain, two domains (flavoHbs) and trHb (truncated Hbs)).

SVM predictions were further analyzed by examination of confusion matrix (CM-model Figure 1) and prediction graphs (Supplementary Figure-2 (a, b, c, d, e), Supplementary Figure-3 (a, b, c, d, e)) [23–25]. According to the prediction score graphs, the negative set (non-HbL) was well separated from the positive sequences. No positive sequence was predicted as negative, and no negative sequence was predicted as positive. For 5-fold cross-validation, 5 modules were constructed for each class. Each module was tested with all bacterial HbL proteins as input to the SVM-classify program. The output of each module was then analyzed, and the best model was selected for confusion matrix and prediction scores graph analysis. A total of 1539 HbL sequences, including 29 sHb, 1402 chimeric (flavoHbs), and 108 truncated Hbs (trHb), were used as input, which was selected in SwissProt/UniProt database. The confusion matrix shows that the SVMs successfully classified all sHb sequences (29/29), but one sequence in both flavoHb and trHb sequences was misclassified with the flavoHb sequence predicted as trHb and the trHb sequence as flavoHb. All other sequences were correctly subclassified (1401/1402 flavoHb, 107/108 trHb) with any method (AC, DC, PSSM, and MM) (Supplementary Table-3). This indicates that the SVM modules developed here are able to recognize and classify HbL sequences with a high prediction rate of almost 100%.

The prediction score graphs for single amino acid composition (AC), dipeptide composition (DC), PSSM, and MM's SVM outputs are presented in Supplementary Figure-2 and Figure-3. In these graphs, the prediction results are represented with positive (HbL prediction) or negative (non-HbL) values. It can be seen in Supplementary Figure-3 (a, b, c, d) that irrespective of the method used almost all non-HbL sequences are correctly predicted as negative by all methods. In hybrid methods recognize the HbL sequences as positive and the non-HbL as negative without any confusion (Supplementary Figure-2e). In subfamily classification, the simplest amino acid composition (AC) and MM methods correctly predicted all sequences, except one flavoHb predicted as trHb and one trHb sequence predicted as flavoHb (Supplementary Figures-3a and d). DC, PSSM, and hybrid methods also correctly predicted both positive and negative sequences, except one sequence present in both classes flavoHb and trHb incorrectly predicted as being negative, (Supplementary Figures-3b, c, and e). This sequence, presented in both datasets (D2UCQ4_XANAP) of flavoHb and trHb, shows in UniProt that hypothetical hemoglobin-like protein HbN (truncated hemoglobin) (Trhbn) (flavohemoglobin). Overall, a large number of sequences were accurately classified with all methods, so the SVM models described here are able to predict a large majority of HbL proteins and classify them properly in their independent classes. In classification also, the SVM models distinguish nearly 100% of the HbL into their subfamily.

### 2.7. ROC Curve Analysis

**Figure 2:**
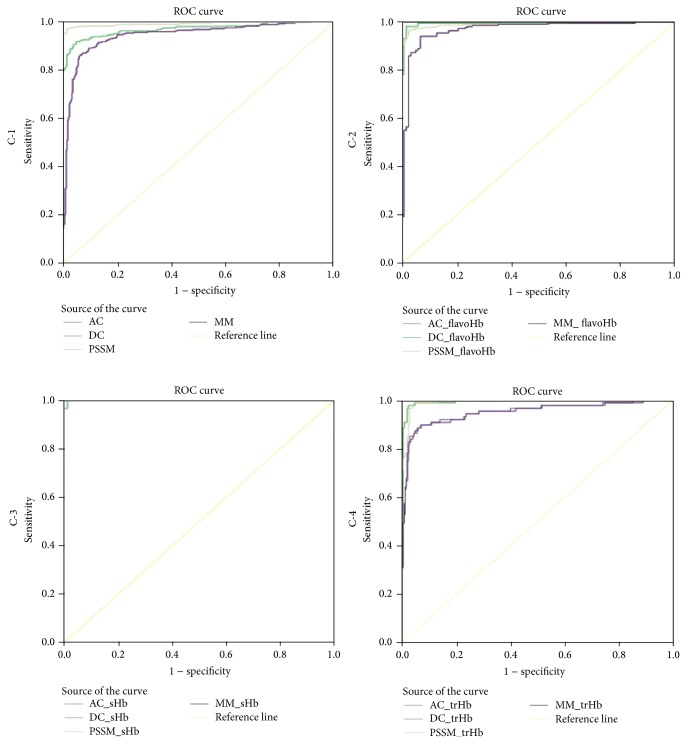
The performance of HbL proteins SVM models by ROC plots. C-1: HbL proteins AUC 0.943, 0.969, 0.992, and 0.943 of AC, DC, PSSM, and MM profile methods, C-2: flavoHbs AUC 0.968, 0.994, 0.991, and 0.968 of AC, DC, PSSM, and MM profile methods, C-3: single-domain (sHb) AUC 1.00, 0.99, 1.00, and 1.00 of AC, DC, PSSM, and MM profile methods, and C-4: trHb AUC 0.950, 0.994, 0.993, and 0.949 of AC, DC, PSSM, and MM profile methods, respectively.

In order to analyze the SVM models developed further, receiver operating characteristic (ROC) plots were produced (Figure 2). The area under curve (AUC) was measured as 0.943, 0.969, 0.992, and 0.943 for HbL models based on AC, DC, PSSM, and MM profile, respectively (Figure 2 C-1). The classification results are shown in Figure 2 C-2 for flavoHbs (AUCs 0.968, 0.994, 0.991, and 0.968), C-3 for single domain (AUCs 1.00, 0.99, 1.00, and 1.00) and C-4 for trHb (truncated Hbs) (AUCs 0.950, 0.994, 0.993, and 0.949). The overall average AUCs were 0.980, 0.997, and 0.972 for flavo, sHb, and trHb and 0.973, 0.975, 0.995, and 0.972 for AC, DC, PSSM, and MM profiles, respectively. Referring to AUC, the DC and PSSM methods are performing slightly better than AC and MM methods. The overall AUC scores show that all methods are predicting BacHbL proteins and their subclasses/families. Thus, the modules developed for all approaches apparently offer good performance for the identification of bacterial HbL sequences.

### 2.8. Comparison with BLAST/PSI-BLAST and Pfam

The foremost and most reliable method used for the characterization of known features in protein sequences is homology based annotation, where a query protein is compared with proteins of known function and the function is assigned only if a query protein is similar to a known target protein [26]. However, homology or similarity based methods fail if the query protein does not possess significant sequence similarity to proteins of known function.

A comparison was constructed using two different ways: BLAST-search and HMM sequence profile from Pfam version 27.0 downloaded on March 18, 2013. In the BLAST-search, an *E*-value cutoff 0.00001 was used against the UniProt/SwissProt database before September 2014 and sequences between 90% and 30% similarity were retained. This filter was only applied for trHb and flavoHb and not for sHb class, due to fewer sequences retrieved. The final BLAST-search results contained 499, 749, and 1203 sequences of sHb, flavoHb, and trHb, respectively. However the sHb BLAST data were included; many flavoHb proteins and the trHb datasets contained some flavoHb sequences. These sequences were tested with our various SVM HbL-models (HbL versus non-HbL; AC, DC, PSSM, and MM models). The results show that 1941 sequences were predicted as positive while 37 sequences were negatively predicted out of 2451 sequences with all approaches (AC, DC, PSSM, and MM). The rest of the sequences (473) were only predicted by either one or two or three approaches. The results from single or combined SVM predictions are summarized, demonstrated in Venn diagram Supplementary Figure 4(A, B, C) of sHb, trHb, and flavoHb, respectively; the complete data is available in Supplementary File-1. The BLAST-search sequences were also analyzed by individual HbL class models (sHb, flavoHb, and trHb) using all approaches (AC, DC, PSSM, and MM). In this case, 103, 589, and 942 sequences were predicted as positive and 359, 1, and 21 sequences were negatively predicted for sHb, flavoHb, and trHb in all approaches. The 359 sHb sequences identified in BLAST-search are mainly flavoHb sequences, so that the sHb models do not classify them as sHb but flavoHb, which indicates that the SVM models are better able to discriminate between sHb and flavoHb related sequences; the summary results are presented in (Venn diagram) Supplementary Figure 4(D, E, F) and the complete prediction data is available as Supplementary File-2. The performance of individual domain prediction is also presented in Supplementary Table-4.

HMM profile is an another more sensitive method in identifying distant homologs; HMM profiles for each of the three individual HbL classes (sHb, flavoHb, and trHb) were constructed and searched against all HMMs profiles of known functional proteins available from the Pfam database at 0.00001 *E*-value. A comparison of the performance of the BLAST-search sequences in Pfam and our SVM models is presented in Table 3. The developed SVM methods also perform similar to Pfam, but no flavoHb proteins were identified as cytochrome reductase domain in Pfam. In our approach, one domain may not be identified by AC, but it can be identified by DC or PSSM or MM. So overall, all HbL proteins can be identified by our methods. In contrast, BacHbpred SVM models were all able to detect this domain.

Finally, to test for the performance of the HbL prediction methodology developed above in the context of full genome prediction, a whole-bacterial genome prediction of the* Bacillus subtilis* having 4053 sequence was conducted. The output results show that 76 proteins were predicted as positive in all approaches. This included protein sequences annotated as HbL (1 sequence) and uncharacterized proteins (11 sequences). The detailed results are shown in Supplementary Figure 4(G) and it was made by Venn diagram. Therefore a majority of positively identified sequences are already annotated while the 11 uncharacterized sequences may present at most 15% of false positive and possibly include a large number of new candidate HbL proteins.

## 3. Conclusion 

In this study, we developed a highly accurate prediction system having several methods to identify bacterial HbL proteins and predict their different classes/families from amino acid sequence data. Using the SVM based prediction approach based on single AC, MM, dipeptide composition (DC), and position specific scoring matrices (PSSM) the technology developed has been shown to provide reasonably high prediction accuracy. Comparative performance analysis of the constructed models indicated that the DC and PSSM methods generally resulted in better prediction than AC and MM on BLAST/Pfam search dataset. Hybrid method, which is the combination of AC and DC, also performs slightly better than DC, showing no much difference in MCC. All of the experimental results, including BLAST/Pfam search dataset, indicate that the proposed HbL prediction tool may be a perspective predictor for the determination of HbL related proteins. Finally, a web server has been developed which will serve the scientific community to identify new HbL proteins and their structural classes. We believe that the developed prediction tool will contribute considerably in providing new directions for the development of such future predictors.

## 4. Material and Methods

### 4.1. Datasets

The original dataset of bacterial HbL proteins was retrieved from UniProt/SwissProt (http://www.uniprot.org) [27] using keyword searches (flavohemoglobin, truncated hemoglobin, and single domain hemoglobin bacteria), resulting in 1539 entries from the organism listed in Supplementary Table-5. This raw dataset included protein sequences annotated as “fragments”, “isoforms”, “potentials”, “similarity”, or “probables” which were removed by a PERL script. A similarity filter (90% similarity cutoff) was also applied that no two sequences have more than 90% similarity. However, this similarity filter was not applied to the single domain hemoglobin (sHb) subset, due to its small size (29 proteins) and relatively high similarity between annotated sHbs.

**Figure 3:**
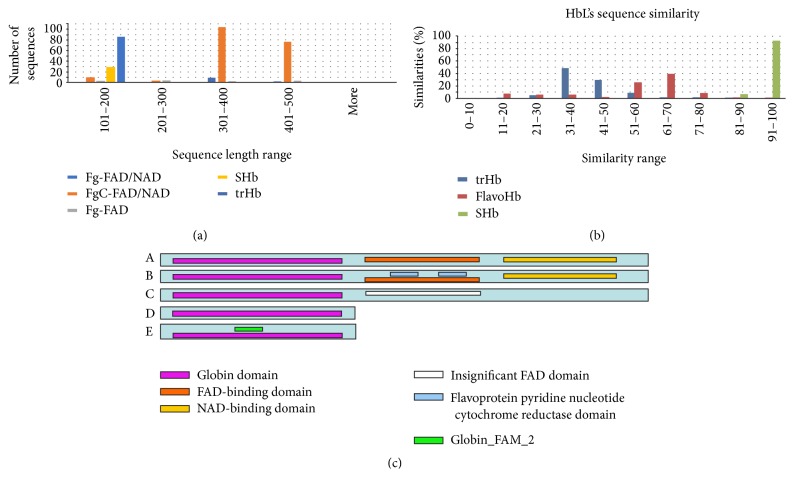
(a) Sequence length histograms of HbL based on domain organization: single domain (sHb), two domains (flavoHbs, i.e., globin-FAD, globin-FAD/NAD, and globin-cyto-FAD/NAD) and trHb (truncated Hbs) (*x*-axis for sequence length range and *y*-axis for number of sequences). (b) Sequence similarity histograms of HbL proteins; single domain (sHb), two domains (flavoHbs) and trHb (truncated Hbs). (c) Domain architecture of HbL protein based on Pfam/InterPro web search tool, (c)(A) flavoHb globin-FAD/NAD, (c)(B) flavoHb globin-cyto-FAD/NAD, (c)(C) flavoHb globin-insignificant-FAD domain, (c)(D) sHb globin domain, and (c)(E) trHb globin_FAM_2 domain.

Curation of the nonredundant dataset for flavohemoglobin (flavoHb) and truncated hemoglobin (trHb) resulted in 217 and 87 peptides filtered from 1343 and 108 SwissProt entries, respectively. The final dataset consisted of 333 high quality bacterial Hbs (HbL) proteins (217 flavoHb +87 trHb +29 sHb) from over 246 bacterial species (180 flavoHb, 64 trHb, and 2 sHb). The sequence length distributions of individual domain of HbL proteins were studied, as shown in Figure 3(a). The longest FgC-FAD/NAD (flavoHb) sequences have a length between 300 and 500 amino acids. In addition, calculations revealed the sequence similarity between all HbL subclasses as shown in Figure 3(b) (MatGAT2.01). In trHb, 50% of the sequences had 31–40% similarity and 30% had a similarity range between 41 and 50%. For the flavoHb subclass, 40% of the sequences had a similarity range between 61 and 70% and 25% of sequences had a similarity between 51 and 60%. The domain architecture of HbL proteins was characterized with Pfam and InterPro tools, and the complete domain organization of HbL is shown in Figure 3(c).

A negative set of 337 nonredundant proteins (90% cutoff), with nearly similar length, was randomly picked from a dataset made by querying SwissProt/UniProt with different keywords and it does not belong to HbL proteins. The similarities between HbL and non-HbL were from 9.67% to 24.71% and the average similarity was 13.78% using Percent Identity Matrix—created by Clustal2.1. The non-HbL sequences are mostly regulatory and proteases; the protein names are, transglycosylase, actin-binding protein, RNA polymerase, phosphate dikinase, pectatelyase, operon regulation, metabolism regulation protein, 5-hydroxytryptamine receptor, osmolarity sensor protein, multiprotein-bridging, mating-type protein, mediator of RNA polymerase, proteasome-interacting protein, RsbT antagonist protein, cyclin-dependent kinase, protein vestigial, synaptobrevin-like protein, mediator of RNA polymerase II transcription, cryptochrome-2, zinc finger CCCH domain, bacterial regulatory proteins,* Salmonella enteritidis*, oxysterol-binding protein, small nuclear ribonucleoprotein, transcriptional activator protein, arginine biosynthesis, bifunctional protein, ubiquitin-protein ligase, putative two-component response regulator, alpha-amylase, angiopoietin-related protein, AMP nucleosidase, glia-derived nexin, hyaluronan-binding protein, hepatocyte growth factor activator, plasma serine protease inhibitor, serine/threonine protein phosphatase, GTPase, Lon protease, pre-mRNA-processing protein, and sporulation kinase.

### 4.2. PSSM Profile

PSSM profiles were developed using the gpsr_1.0 package, which is freely available for Linux/Windows (http://www.imtech.res.in/raghava/gpsr/), run against the nonredundant (nr) database downloaded through NCBI (ftp://ftp.ncbi.nih.gov/blast/db/). The position specific scoring matrix was calculated using the suite (GPSR) programs. In the development of PSSM profile, seq2pssm_imp, pssm_comp, and col2svm programs were used to generate the SVM_light input format (a 400-point vector representing the substitution rate of each amino acid into any other) [28–31]. 

*seq2pssm_imp: To Calculate PSSM Matrix in Column Format without Any Normalization*. seq2pssm_imp was used to calculate the PSSM matrix in column format without any normalization, by performing PSI-BLAST-searches against the nonredundant protein database using different iterations (e.g., 3) with a cutoff *E*-value 0.001. For a sequence of length *N*, an *N* × 20 position specific substitution matrix (*m*) was computed from the PSI-BLAST alignment output where *m* [*i*, *j*] provided information on the evolutionary conservation of residue type (*j*) at sequence position (*i*). The values of PSSM matrix vary within a large range, which makes it difficult to run SVM. Thus, every PSSM element *X*(*i*) at position (*i*) is normalized using the program pssm_n2 based on the following formula:

(1) $$\begin{array}{c} X\left(\;i\;\right)=\frac{\left(n\left(i\right)-l\left(i\right)\right)}{\left(m\left(i\right)-l\left(i\right)\right)}, \end{array}$$

where *X*, *n*, *l*, and *m* are, respectively, defined as the normalization value, the residue actual position score, the minimum score, and the maximum score of the PSSM outputs for a single residue position. Here (*i*) is defined as the residue's position. For example, if the PSSM output for a single position is {−279, −326, −515, −410, −186, −484, −373, 101, −346, 99, 918, −430, −450, −256, −349, −351, −250, 114, −352, −293}, then −515 and 918 are the minimum and maximum scores. After normalization by the above formula, the first score will be normalized to ((−279)−(−515))/(918 − (−515)) = 236/1433 = 0.1646, and the position scores vector will be converted to {0.1646, 0.1318, 0, 0.0732, 0.2295, 0.0216, 0.0990, 0.4298, 0.1179, 0.4284, 1, 0.0593, 0.0453, 0.1807, 0.1158, 0.1144, 0.1849, 0.4389, 0.1137, 0.1549}. The values are now normalized between 0 and 1, so that the minimum scores receive “0” and the maximum scores are set to “1.” 

*pssm_comp: To Compute PSSM Composition (400 Points)*. The pssm_comp program is used to calculate the PSSM composition in a vector of 400 dimensions, by computing the composition of occurrences of each type of amino acids corresponding to each type of amino acids present in protein sequence. According to this statement, each column has 20 values instead of one. Every element of this input vector was subsequently divided by the length of the sequence. The resultant matrix with 400 elements was used as an input feature for running SVM. 

*col2svm: To Generate SVM_light Input Format*. The col2svm program is used to convert the PSSM normalization output file to composition format file, which is used for running SVM training. Mainly this program is used to assign the (+ve) label for positive sequences and (−ve) label for negative sequences.

### 4.3. Amino Acid Composition (AC)

Amino acid composition is the fraction of each amino acid in a protein. The fraction of all 20 natural amino acids was calculated using

(2) Fraction  of  amino  acidi=Total  number  of  amino  acidiTotal  number  of  amino  acids  in  protein,

where (*i*) can be any amino acid.

### 4.4. Dipeptide Composition (DC)

Dipeptide composition is used to encapsulate the global information about each protein sequence, which gives a fixed pattern length of 400 (20 × 20). The fraction of each dipeptide was calculated using

(3) Fraction  of  depi+1=Total  number  of  depi+1Total  number  of  all  possible  dipeptides,

where dep(*i* + 1) is one out of 400 dipeptides.

### 4.5. Amino Acid Composition Feature Vectors

The average amino acid composition (AC) was calculated using an alphabetical ordering of the amino acids “ACDEFGHIKLMNPQRSTVWY.” The MM (maximum to minimum) composition vector was obtained by sorting the average HbL sequences AC composition from the most abundant to the less abundant amino acids. The residues order for MM is “ALEKIVGDPNFQRTMYSHWC” and it was used as a fixed vector to calculate the MM profile.

### 4.6. SVM

Support vector machine (SVM) is a commonly used tool to solve two-class classification problems. It has been shown to be an effective method in computational biology. In this study, we used a free downloadable package: SVMlight, available at http://svmlight.joachims.org [32–34]. The SVM training was carried out by optimization of various kernel function parameters and the value of the regularization parameter *C*.

### 4.7. Confusion Matrix

A confusion matrix (also known as the contingency matrix) contains information about actual and predicted classifications done by a classification system as illustrated in Supplementary Figure-2. The performance of such systems is commonly evaluated using the data in the matrix. In the confusion matrix it is easy to see if the system is confusing two classes. When a dataset is unbalanced (when the number of samples in different classes varies greatly) the error rate of the classifier is not representative of the true performance, and the confusion matrix needs a more detailed analysis.

### 4.8. Evaluation of Performance

The performance was evaluated by 5-fold cross-validation. The whole dataset was randomly divided into five sets of approximately equal size. Four sets were used for training and one set was used for testing. Different sets were chosen for 5-fold assessment one by one. The results from the classification were estimated by different measures: accuracy (ACC), sensitivity (SN), specificity (SP), and Matthews correlation coefficient (MCC). Accuracy is the percentage of correctly predicted positive and negative examples. Sensitivity is the percentage of positive examples (HbL proteins), which are correctly predicted as positive. Specificity is the percentage of negative examples (non-HbL proteins), which are correctly predicted to be negative. MCC is a measure of the quality of a binary classification system. The following equations were used:

(4) AccuracyACC=TP+TNTP+TN+FP+FN,SensitivitySN=TPTP+FN,SpecificitySP=TNTN+FP,MCC=TP×TN−FP×FNTP+FPTP+FNTN+FPTN+FN,FPR=FPFP+TN,

where TP, TN, FP, and FN are the numbers of true positive, true negative, false positive, and false negative residues of the prediction, respectively. Sensitivity and specificity are used to plot receiver operating characteristic (ROC) curves to calculate the AUC.

The aim of this work is to propose a new predictor for HbL protein and its subclasses determination based on features such as amino acid composition (AC), dipeptide composition (DC), hybrid approach (combination of AC and DC), and evolutionary information (i.e., PSSM profile). The SVM method was extended to develop a new approach for protein prediction based on max to min residues profiling. To achieve the aim, firstly, we constructed HbL protein dataset that consist of three main classes, that is, single domain hemoglobin (sHb), truncated hemoglobin (trHb), and flavohemoglobin (flavoHb). Further, HbL proteins can also be classified according to their domain architecture such as globin-sHb, flavoglobin, flavoglobin-FAD-binding domain, flavoglobin-FAD/NAD-binding with cytochrome reductase domain, and truncated hemoglobin (trHb). A negative non-HbL protein dataset was also constructed and used as an extra class for background controls. The performance of the prediction modules developed for bacterial HbL proteins was analyzed by both cross-validation and confusion matrix analysis. Furthermore, the SVM based approached was compared to homology detection methods such as BLAST/Pfam domain search. Initially, we did blast locally to all sequences (sHb, flavoHb, and trHb), then collected the IDs, and retrieved the sequences in UniProt/SwissProt database; for reducing the dataset size, we have chosen 90% cutoff. In the collected datasets, we found that the sequence similarity was between 30 and 90% for flavoHb and trHb proteins, but all sHb proteins available were used to run BLAST-search, due to the small number of known sHb proteins.

### 4.9. Web Server

In this study, we have developed an online server BacHbpred implemented on the World Wide Web (WWW), which is freely accessible at http://mamsap.it.deakin.edu.au/bac_hbpred/home.html. All the scripts of the methods are written in CGI-PERL program and the interface is designed in HTML (Hypertext Manipulation Language). The server provides a user-friendly interface and allows users to submit their query sequences and the results are displayed in a simple tabular format.

## Supplementary Material

Supplementary Figure 1: 1a) Distribution of amino acids in bacterial HbL proteins and non-HbL; 1b) amino acid distribution in bacterial HbL proteins (single domain, flavoHb and TrHb)
Supplementary Figure 2: Prediction score graphs for the performance of models which developed by HbLs and non-HbLs sequences. a). AC model performance on HbL and non-HbL sequences , b). DC model performance on HbL and non-HbL sequences, c). PSSM model performance on HbL and non-HbL sequences, d). MM model performance on HbL and non-HbL sequences and e). Hybrid model performance on HbL and non-HbL sequences. The best model was used to generate the results as well as for implementation in our online server (X-axis is indexed on HbL proteins and the Y-axis is the score of the prediction).
Supplementary Figure 3: Prediction score graphs for the performance of subfamilies model on their dataset sequences. a). AC subfamilies model performance on sHb, flavoHb and trHb sequences, b). DC subfamilies model performance on sHb, flavoHb and trHb sequences, c). PSSM profile based subfamilies model performance on sHb, flavoHb and trHb sequences, d). MM profile based subfamilies model performance on sHb, flavoHb and trHb sequences, e). Hybrid approach based subfamilies model on sHb, flavoHb and trHb sequences. The X-axis is indexed on HbL proteins (SHb, FlavoHb and trHb in respective order) and the Y-axis is the score of the prediction.
Supplementary Figure 4: Venn diagram summarizing the BLAST-search data was performed by the proposed HbL and Non-HbL developed models (AC, DC, PSSM, MM). A) All models performance on sHb BLAST-search sequences, B) All models performance on trHb BLAST-search sequences, C) All models performance on flavoHb BLAST-search sequences, D). sHb's individual models of all methods prediction performance on sHb BLAST-search sequences, E). trHb's all models performance on trhb sequences, F). flavoHb's all methods models on flavoHb sequences and G). genome level prediction of Bacillius subtilies sequences by the developed (AC, DC, PSSM, MM) models.
Supplementary Table 1: Performance of various SVM modules average accuracy and standard deviation (SD) of all approaches and HbLs classifications (sHb, flavoHb and truncated Hbs) developed using amino acid (AC), dipeptide composition (DC), PSSM profile and MM profile respectively.
Supplementary Table 2: Performance of various SVM modules average accuracy and standard deviation (SD) of HbLs domains classifications (flavo-globin, flavo-globin-cyto-FAD/NAD, flavo-glob-FAD, single (SHb) and trHb domain) developed using amino acid (AC), dipeptide composition (DC), PSSM and MM profile respectively.
Supplementary Table 3: Confusion matrix results of all Bac-HbL proteins (SHb, FlavoHb, TrHb). Results obtained with the best models, which have been implemented in the Bac-Hbpred server.
Supplementary Table 4: Prediction based on BLAST-search dataset for individual domain of HbL Protein in all approaches. C for correct prediction i.e same sequence predict in one and other models. I-C for incorrect prediction i.e not predicted in a single as well as in other models.
Supplementary Table 5: Organism list of HbL Proteins( flavoHb,trHb and sHb).
Supplementary file-1: Prediction performance of BLAST-search dataset by BacHbpred's AC, DC,PSSM and MM approaches. Green color – same sequence predicted in all models, Red color- not predicted in all models.
Supplementary file-2: Prediction performance of BLAST-search dataset by BacHbpred's individual models (classification models) sHb, FlavoHb, trHb in all approaches (AC, DC, PSSM and MM). Green color – same sequence predicted in all models, Red color- not predicted in all models.
Supplementary file-3: Prediction performance of the developed all approached models (AC, DC, PSSM and MM) on Bacillius subtilies sequences. The detailed information of the positively predicted 76 sequences are listed.

## Figures and Tables

**Table 1:** Performance of various SVM modules of HbL proteins predictions with non-HbL and HbL classification (single domain, two domains (flavoHbs) and truncated Hbs (trHb)) developed using various methods: amino acids (AC), dipeptides (DC), PSSM, and MM profiles.

	Methods	ACC	SN	SP	MCC	Parameter	
						*γ*	*C*
HbL versus non-HbL	AC	86.14	96.18	76.11	0.82	25	400
	DC	83.02	94.78	71.27	0.78	1	375
	PSSM	90.20	97.76	82.64	0.89	1	300
	MM	86.28	96.08	76.49	0.83	25	450
	Hybrid	85.21	95.80	74.62	0.81	0.1	375

*sHb*	AC	94.96	100	94.56	0.97	15	9
	DC	83.23	100	82.15	0.91	0.2	250
	PSSM	95.05	100	94.66	0.97	5	7
	MM	94.87	100	94.46	0.97	1	150
	Hybrid	91.51	100	90.83	0.95	0.1	350

*FlavoHb*	AC	96.46	100	89.67	0.95	10	300
	DC	87.50	100	63.58	0.80	1	350
	PSSM	95.05	100	85.59	0.93	1	350
	MM	96.46	100	89.67	0.95	10	300
	Hybrid	90.29	100	71.73	0.84	1	150

*trHb*	AC	85.26	98.89	80.62	0.89	5	350
	DC	78.17	98.89	71.13	0.83	1	275
	PSSM	87.97	100	83.88	0.92	1	400
	MM	85.07	99.26	80.25	0.89	4	500
	Hybrid	80.03	100	73.25	0.85	1	150

**Table 2:** Performance of various SVM modules of HbL proteins domains classifications (flavoglobin, flavoglobin-cyto-FAD, flavoglobin-FAD, and single and trHb domain) developed using amino acid (AC), dipeptide composition (DC), PSSM, and MM profile, respectively.

HbL protein domain	Methods	ACC (%)	SN (%)	SP (%)	MCC	Parameters	
						*γ*	*C*
Flavoglobin (FAD-insignificant)	AC	91.88	65.62	92.69	0.74	50	200
	DC	78.09	56.25	78.75	0.52	2	250
	PSSM	84.05	78.13	84.23	0.77	2	400
	MM	93.28	100.00	93.08	0.96	25	450
	Hybrid	82.00	62.50	82.59	0.62	1	450

Flavoglobin-cyto-FAD/NAD	AC	89.65	99.51	75.89	0.86	10	200
	DC	87.78	98.55	72.77	0.83	5	200
	PSSM	91.04	100	78.57	0.89	2	500
	MM	89.17	96.96	78.34	0.84	25	400
	Hybrid	89.00	98.56	75.67	0.85	3	350

Flavoglobin-FAD	AC	83.68	50.00	84.71	0.53	10	275
	DC	77.05	59.37	77.60	0.54	1	275
	PSSM	82.74	18.75	84.71	0.09	1	500
	MM	89.74	50.00	90.96	0.60	15	500
	Hybrid	76.77	43.75	77.78	0.37	1	200

Single bac domain (globin-like)	AC	94.96	100	94.56	0.97	15	9
	DC	83.23	100	82.15	0.91	0.2	250
	PSSM	95.05	100	94.66	0.97	5	7
	MM	94.87	100	94.46	0.97	1	150
	Hybrid	91.51	100	90.83	0.95	0.1	350

Truncated BacHb domain (globin_trunc_bac-like)	AC	85.26	98.89	80.62	0.89	5	350
	DC	78.17	98.89	71.13	0.83	1	275
	PSSM	87.97	100	83.88	0.92	1	400
	MM	85.07	99.26	80.25	0.89	4	500
	Hybrid	80.03	100	73.25	0.85	1	150

**Table 3:** HbL domain prediction performance of BLAST-search sequences compared with Pfam along with BacHbpred all models (AC, DC, PSSM, and MM).

	Total	Pfam	AC	DC	PSSM	MM
SHb	499	162	140	140	103	140
Flavoglobin	749	04	31	27	07	48
Flavoglobin-cyto-FAD/NAD	749	673^*∗*^	667	605	578	631
Flavoglobin-FAD	749	30	30	20	00	34
trHb	1203	1130	1008	1081	1164	1011

^*∗*^Pfam predicts flavoglobin with FAD/NAD only, but it does not show any signal for cytochrome reductase domain.
