# Supplementary material for: BacHbpred: Support Vector Machine Methods for the Prediction of Bacterial Hemoglobin-Like Proteins
Source: Adv Bioinformatics. 2016 Feb 29;2016:8150784. doi: 10.1155/2016/8150784 (PMC4789356; doi:10.1155/2016/8150784)
Supplement: Supplementary file 1 — Supplementary Figure 1: 1a) Distribution of amino acids in bacterial HbL proteins and non-HbL; 1b) amino acid distribution in bacterial HbL proteins (single domain, flavoHb and TrHb) Supplementary Figure 2: Prediction score graphs for the performance of models which developed by HbLs and non-HbLs sequences. a). AC model performance on HbL and non-HbL sequences , b). DC model performance on HbL and non-HbL sequences, c). PSSM model performance on HbL and non-HbL sequences, d). MM model performance on HbL and non-HbL sequences and e). Hybrid model performance on HbL and non-HbL sequences. The best model was used to generate the results as well as for implementation in our online server (X-axis is indexed on HbL proteins and the Y-axis is the score of the prediction). Supplementary Figure 3: Prediction score graphs for the performance of subfamilies model on their dataset sequences. a). AC subfamilies model performance on sHb, flavoHb and trHb sequences, b). DC subfamilies model performance on sHb, flavoHb and trHb sequences, c). PSSM profile based subfamilies model performance on sHb, flavoHb and trHb sequences, d). MM profile based subfamilies model performance on sHb, flavoHb and trHb sequences, e). Hybrid approach based subfamilies model on sHb, flavoHb and trHb sequences. The X-axis is indexed on HbL proteins (SHb, FlavoHb and trHb in respective order) and the Y-axis is the score of the prediction. Supplementary Figure 4: Venn diagram summarizing the BLAST-search data was performed by the proposed HbL and Non-HbL developed models (AC, DC, PSSM, MM). A) All models performance on sHb BLAST-search sequences, B) All models performance on trHb BLAST-search sequences, C) All models performance on flavoHb BLAST-search sequences, D). sHb's individual models of all methods prediction performance on sHb BLAST-search sequences, E). trHb's all models performance on trhb sequences, F). flavoHb's all methods models on flavoHb sequences and G). genome level prediction of Bacilli [file 8150784.f1.pdf]

supplementary figure - 1

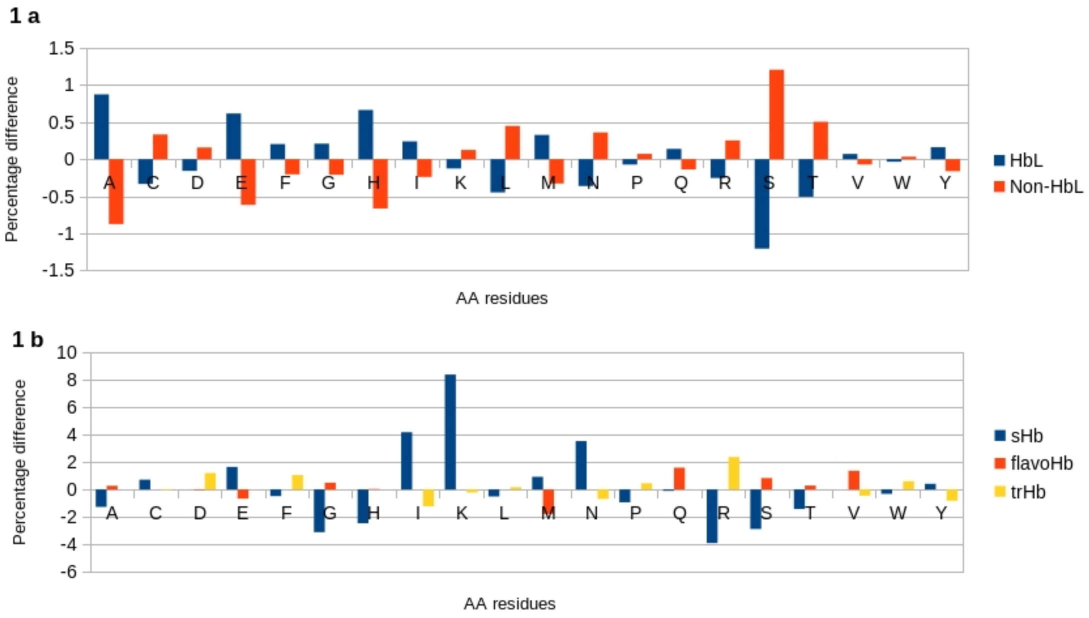

supplementary figure - 2

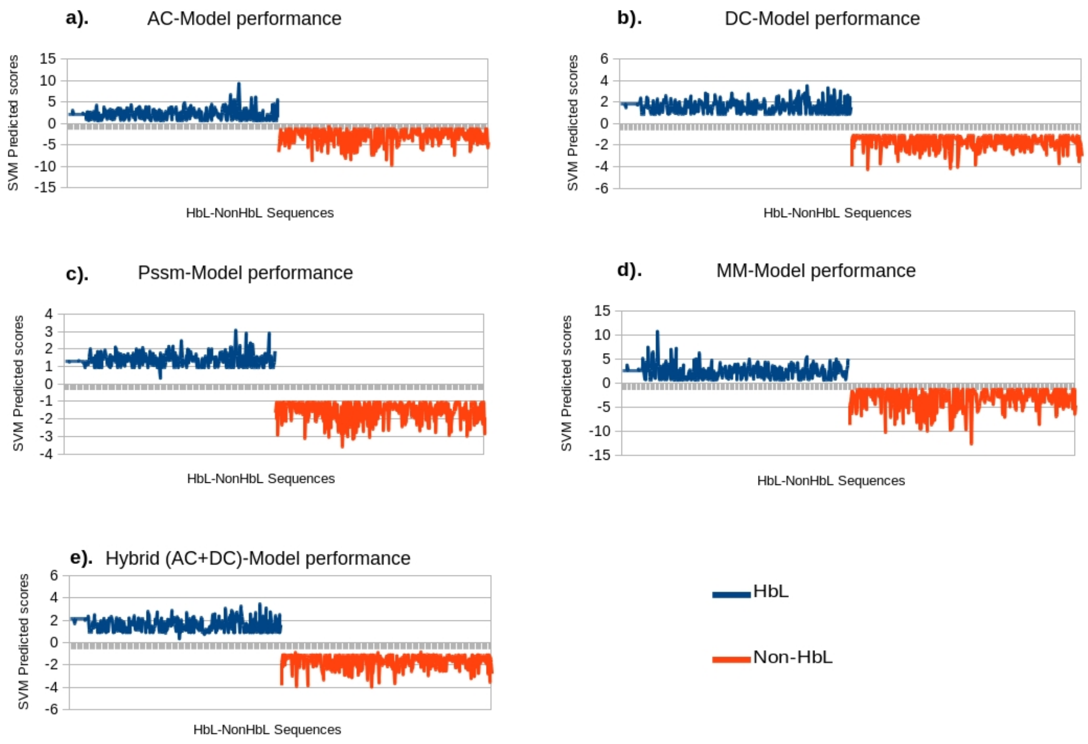

supplementary figure - 3

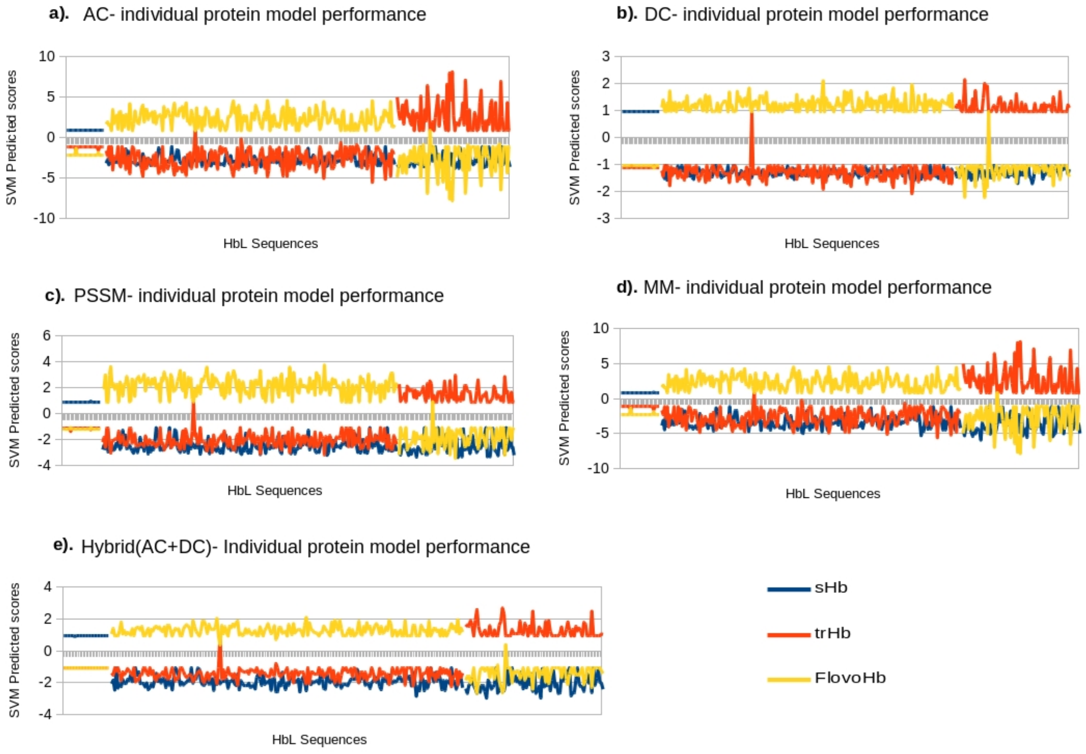

supplementary figure – 4

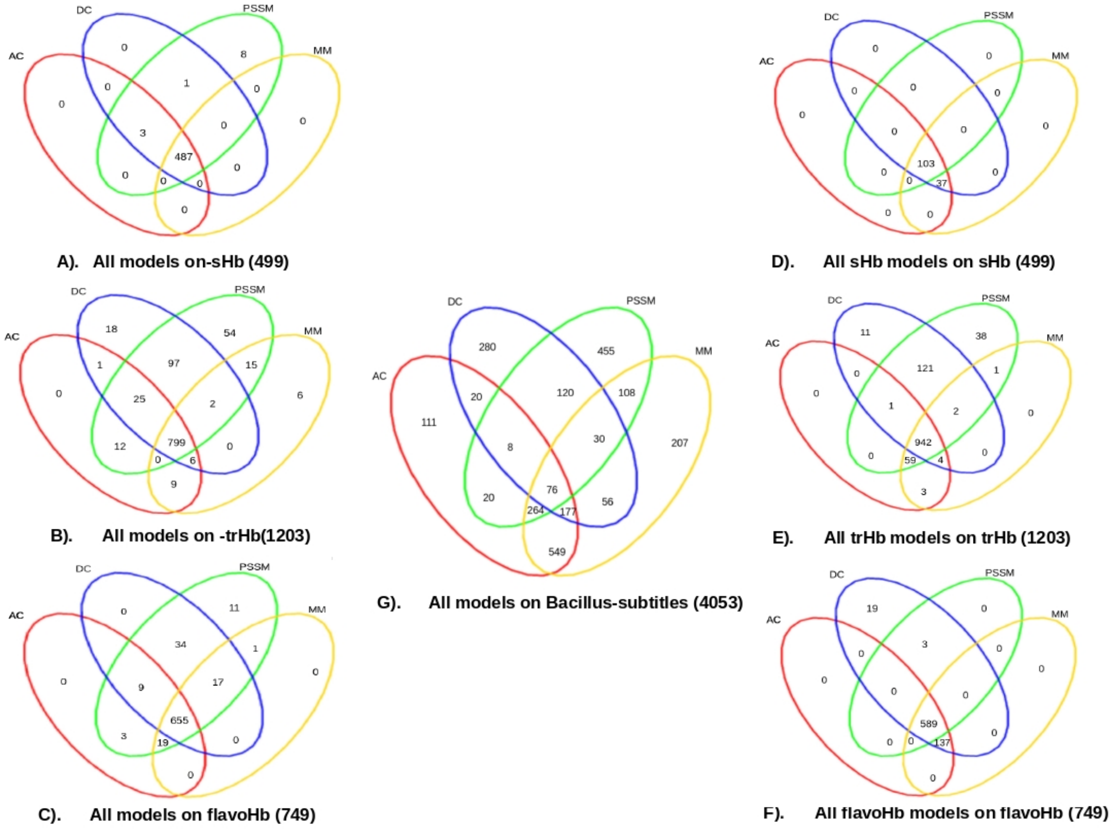

Supplementary Table-1

		AC	DC	PSSM	MM	Hybrid
**All – approaches**	Avg-ACC	85.76	80.45	87.93	85.46	82.39
	STDEV (SD)	1.59	6.63	4.89	2.64	5.66
**SHb**	Avg-ACC	94.04	72.98	93.72	94.99	88.22
	STDEV (SD)	2.59	23.17	3.19	2.53	7.76
**Flavo Hb**	Avg-ACC	95.65	83.92	93.08	95.65	87.53
	STDEV (SD)	0.92	7.93	4.14	0.92	6
**TrHb**	Avg-ACC	82.66	70.67	85.17	82.38	73.71
	STDEV (SD)	6.07	15.94	6.5	6.33	13.89

Supplementary Table-2

HbL domains		AC	DC	PSSM	MM	Hybrid
Flavo globin (FAD-insignificant)	Avg-ACC	90.07	70.65	80.9	91.97	75.63
	STDEV (SD)	4.41	17.87	9.02	3.61	15.2
Flavo globin_cyto_FAD/NAD	Avg-ACC	88.67	85.77	89.01	87.59	87.29
	STDEV (SD)	1.36	2.55	2.8	2.07	2.24
Flavo globin_FAD	Avg-ACC	79.24	69.94	78.44	88.26	70.93
	STDEV (SD)	10.9	17.43	10.91	4.56	15.18
Shb (Globin_like)	Avg-ACC	94.04	72.98	93.72	94.99	88.22
	STDEV (SD)	2.59	23.17	3.19	2.53	7.76
TrHb (Globin_trunc_bac-like)	Avg-ACC	82.66	70.67	85.17	82.38	73.71
	STDEV (SD)	6.07	15.94	6.5	6.33	13.89

Supplementary Table – 3

	Total	*S-Hb*				*FlavoHb*				*trHb*			
Methods		AC	DC	PSSM	MM	AC	DC	PSSM	MM	AC	DC	PSSM	MM
S-Hb	29	29	29	29	29	0	0	0	0	0	0	0	0
trHb	108	0	0	0	0	1	1	1	1	107	107	107	107
FlavoHb	1402	0	0	0	0	1401	1401	1401	1401	1	1	1	1

AC - Amino acid composition, DC- dipeptide composition, PSSM- position specific scoring matrix, MM- Max to Min profile. S_Hb- single domain, Flavo Hb FlavoHemoglobin, trHb truncated Hemoglobin, ACC- accuracy, SN- Sensitivity, SP- specificity, MCC- Matthews correlation coefficient, C- tradeoff value, γ- gamma factor (a parameter in RBF kernel).

Supplementary Table-4

	AC		DC		PSSM		MM	
	C	I-C	C	I-C	C	I-C	C	I-C
**SHb**	140	359	140	359	140	359	140	359
**Flovo-globin**	31	718	27	722	7	742	48	701
**Flovo-globin-cyto-FAD/NAD**	667	82	605	144	577	172	630	119
**Flovo-globin-FAD**	30	719	19	730	0	749	33	716
**trHb**	922	281	1000	203	1124	79	959	244

C- correct prediction,   I-C Incorrect predictions

FlavoHb	*FlavoHb*	*FlavoHb*	*trHb*	*sHb*
Achromobacter arsenitoxydans(1)	Escherichia coli (2)	Psychromonas sp. (1)	Acidiphilium cryptum (1)	Campylobacter jejuni subsp(28)
Achromobacter piechaudii (2)	Escherichia hermannii (1)	Rahnella aquatilis (1)	Aequorivita sublithincola(1)	Campylobacter coli(1)
Achromobacter xylosoxidans(1)	Ferrimonas balearica (1)	Ralstonia eutropha(1)	Arthrobacter aurescens (1)	
Acinetobacter baumannii (1)	Francisella philomiragia subsp.(1)	Ralstonia metallidurans(1)	Arthrobacter chlorophenolicus(1)	
Acinetobacter sp. (1)	Frankia alni (2)	Rhizobium meliloti (1)	Arthrospira sp. (1)	
Advenella kashmirensis(1)	Geobacillus sp.(1)	Rhizobium sp. (2)	Azoarcus sp.(1)	
Aeromonas salmonicida subsp.(1)	Geobacillus thermodenitrificans (1)	Rhodopirellula baltica (1)	Azospira oryzae (1)	
Aeromonas veronii (1)	Geobacillus thermoglucosidans(1)	Salmonella enterica subsp.(1)	Bacillus cereus(1)	
Alcaligenes bronchisepticus(1)	Geobacillus thermoleovorans (1)	Serratia odorifera (1)	Bacillus licheniformis (1)	
Alicyclobacillus acidocaldarius (1)	Glaciecola agarilytica (1)	Serratia proteamaculans (1)	Bacillus subtilis (1)	
Alicyclobacillus hesperidum (1)	Glaciecola mesophila (1)	Serratia symbiotica str. (1)	Belliella baltica (3)	
Aliivibrio salmonicida (1)	Grimontia hollisae (1)	Shewanella baltica (1)	Brachybacterium faecium (1)	
Azoarcus sp.(1)	Hahella chejuensis (1)	Shewanella piezotolerans (1)	Brachyspira hyodysenteriae(1)	
Azotobacter vinelandii (1)	Halobacillus halophilus (1)	Shewanella sp.(2)	Brachyspira murdochii (1)	
Bacillus alcalophilus (1)	Helicobacter canadensis(1)	Shewanella violacea (1)	Brachyspira pilosicoli (1)	
Bacillus amyloliquefaciens (1)	Herbaspirillum seropedicae (1)	Shewanella woodyi (1)	Bradyrhizobium sp. (4)	
Bacillus atrophaeus (1)	Herminiimonas arsenicoxydans(1)	Sinorhizobium medicae (1)	Brevibacillus laterosporus (1)	
Bacillus cereus(4)	Klebsiella oxytoca (1)	Sporosarcina newyorkensis(1)	Burkholderia sp.(2)	
Bacillus clausii(1)	Klebsiella pneumoniae subsp. (1)	Staphylococcus aureus subsp.(1)	Campylobacter jejuni subsp. (1)	
Bacillus coagulans (1)		Streptomyces venezuelae(2)	Clavibacter michiganensis subsp. (1)	
Bacillus halodurans (1)	Klebsiella sp. (1)	Thioalkalivibrio sp. (1)	cluster alpha proteobacterium (1)	
Bacillus methanolicus(1)	Lentisphaera araneosa (1)	Thiobacillus denitrificans (1)	Cupriavidus necator(2)	
Bacillus mycoides(1)	Lyngbya sp. (1)	Tolumonas auensis (1)	Flexibacter litoralis(1)	
Bacillus pseudofirmus(1)	Lysinibacillus sphaericus (1)	Vibrio brasiliensis (1)	Geobacillus sp.(1)	
Bacillus smithii (1)	Marinomonas sp. (1)	Vibrio cholerae serotype(1)	Hahella chejuensis (2)	
Bacillus sp. (2)	Methylobacterium extorquens (1)	Vibrio coralliilyticus (1)	Herbaspirillum seropedicae(1)	
Bacillus subtilis (9)		Vibrio fischeri (1)	Hyphomicrobium denitrificans(1)	
Beggiatoa sp. (1)	Methylobacterium sp.(1)	Vibrio furnissii (1)	Hyphomicrobium sp. (1)	
Bordetella avium(1)	Methylophaga sp. (2)	Vibrio harveyi (1)	Kytococcus sedentarius (1)	
Bordetella bronchiseptica (1)	Methyloversatilis universalis (1)	Vibrio mimicus (1)	Maricaulis maris (1)	
Bordetella petrii (1)	Micavibrio aeruginosavorus (1)	Vibrio nigripulchritudo(1)	Methylomicrobium alcaliphilum (2)	
Brevibacillus laterosporus (1)	Morganella morganii subsp. (1)	Vibrio parahaemolyticus (2)	Methylomonas sp.(3)	
Brevibacillus sp.(2)	Mycobacterium smegmatis (1)	Vibrio shilonii (1)	Micrococcus luteus(1)	
Brevundimonas diminuta (1)	Nitrobacter sp.(1)	Vibrio sinaloensis(1)	Microcystis aeruginosa(1)	
Burkholderia cenocepacia (1)	Nitrococcus mobilis (1)	Vibrio sp. (2)	Micromonospora lupini str. (1)	
Burkholderia graminis (1)	Oceanimonas sp. (1)	Vibrio splendidus(1)	Mycobacterium bovis (2)	
Burkholderia mallei (1)	Oceanobacillus iheyensis (1)	Vibrio tubiashii (1)	Mycobacterium chubuense(3)	
Burkholderia multivorans (1)	Paenibacillus curdlanolyticus (1)	Vibrio vulnificus (1)	Mycobacterium gilvum(1)	
Burkholderia sp.(7)	Paenibacillus dendritiformis(1)	Waddlia chondrophila(1)	Mycobacterium leprae(1)	
Burkholderia terrae(2)	Paenibacillus lactis (1)	Xanthomonas albilineans(2)	Mycobacterium paratuberculosis(1)	
Burkholderia vietnamiensis(1)	Paenibacillus mucilaginosus(1)	Xenorhabdus bovienii (1)	Mycobacterium rhodesiae (4)	
Caldalkalibacillus thermarum (1)	Paenibacillus sp. (3)	Xenorhabdus nematophila (1)	Mycobacterium vanbaalenii(1)	
Candidatus Burkholderia(1)	Pantoea ananatis (1)	Xylella fastidiosa (1)	Nostoc commune(1)	
Caulobacter sp. (1)	Pantoea sp. (3)	Yersinia aldovae (1)	Novosphingobium sp. (1)	
Chromobacterium violaceum (1)	Pantoea stewartii subsp.(1)	Yersinia bercovieri (1)	Ornithobacterium rhinotracheale(1)	
Citrobacter rodentium (1)	Pantoea vagans (1)	Yersinia enterocolitica (1)	Owenweeksia hongkongensis(1)	
Commensalibacter intestini(1)	Pasteurella multocida subsp. (1)	Yersinia intermedia (1)	Parachlamydia acanthamoebae (1)	
Crocosphaera watsonii(1)	Pectobacterium atrosepticum(1)	Yersinia pestis(1)	Pelagibacterium halotolerans (1)	
Cronobacter turicensis (1)	Photobacterium angustum (1)	Yersinia ruckeri (1)	Phenylobacterium zucineum (2)	
Cupriavidus necator (1)	Photobacterium damselae subsp. (1)	Yokenella regensburgei(1)	Pseudoalteromonas haloplanktis(1)	
Deinococcus proteolyticus (1)			Pseudoalteromonas sp.(3)	
Deinococcus radiodurans (1)	Photobacterium leiognathi subsp. (1)		Rheinheimera nanhaiensis (1)	
Desmospora sp. (1)	Photobacterium profundum (1)		Rhizobium sp.(1)	
Dickeya dadantii (2)	Photorhabdus luminescens subsp.(1)		Riemerella anatipestifer (1)	
Dickeya zeae (1)	Planctomyces brasiliensis(1)		Saccharomonospora azurea(1)	
Dinoroseobacter shibae(2)	Planococcus antarcticus(2)		Saccharomonospora viridis (1)	
Edwardsiella tarda(2)	Planococcus donghaensis(1)		Sanguibacter keddieii (1)	
Enterobacter cancerogenus (1)	Proteus mirabilis (1)		SAR116 cluster alpha	
[illegible]	[illegible]		[illegible]	

Supplementary Table-5
